# Spatial Metrics of Interaction between CD163-Positive Macrophages and Cancer Cells and Progression-Free Survival in Chemo-Treated Breast Cancer

**DOI:** 10.3390/cancers14020308

**Published:** 2022-01-08

**Authors:** Brenton A. Maisel, Misung Yi, Amy R. Peck, Yunguang Sun, Jeffrey A. Hooke, Albert J. Kovatich, Craig D. Shriver, Hai Hu, Marja T. Nevalainen, Takemi Tanaka, Nicole Simone, Li Lily Wang, Hallgeir Rui, Inna Chervoneva

**Affiliations:** 1 Department of Pharmacology and Experimental Therapeutics, Thomas Jefferson University, Philadelphia, PA 19107, USA; maiselbr@gmail.com (B.A.M.); Misung.Yi@jefferson.edu (M.Y.); 2 Department of Pathology, Medical College of Wisconsin, Milwaukee, WI 53226, USA; apeck@mcw.edu (A.R.P.); ysun@mcw.edu (Y.S.); mnevalainen@mcw.edu (M.T.N.); 3 John P. Murtha Cancer Center, Uniformed Services University and Walter Reed National Military Medical Center, Bethesda, MD 20814, USA; jhooke@hjfresearch.org (J.A.H.); akovatich@hjfresearch.org (A.J.K.); craig.d.shriver.civ@mail.mil (C.D.S.); 4 Chan Soon-Shiong Institute of Molecular Medicine at Windber, Windber, PA 15963, USA; H.Hu@wriwindber.org; 5 Department of Pathology, University of Oklahoma Health Sciences Center, Stephenson Cancer Center, Oklahoma City, OK 73104, USA; Takemi-Tanaka@ouhsc.edu; 6 Department of Radiation Oncology, Thomas Jefferson University, Philadelphia, PA 19107, USA; Nicole.Simone@jefferson.edu; 7 Department of Translational Hematology and Oncology Research, Cleveland Clinic Foundation, 9500 Euclid Avenue, Cleveland, OH 44195, USA; WANGL9@ccf.org

**Keywords:** tumor-associated macrophages, cancer biomarkers, tumor immune microenvironment, spatially-resolved immunohistochemistry data, marked point patterns, nearest neighbor distance, microscopic image analysis, quantitative pathology, spatial interactions, breast cancer

## Abstract

**Simple Summary:**

A majority of breast cancer deaths are caused by aggressive molecular subtypes that are at high risk of progression. Patients with high-risk breast cancer commonly receive first-line systemic chemotherapy. Chemotherapy exerts direct cytotoxic effects on proliferating cancer cells. In addition, significant effects of chemotherapy are mediated through immune-boosting anti-cancer mechanisms that counteract immunosuppressive tumor-associated macrophages (TAMs). The aim of our study was to evaluate the potential prognostic value of the prevalence and the spatial localization of CD$163^{+}$ TAMs in tumor tissue from breast cancer patients treated with chemotherapy after surgery. We developed a novel algorithm that identifies CD$163^{+}$ TAMs in an objective manner and quantifies spatial interactions between CD$163^{+}$ TAMs and cancer cells using distance-based metrics. Our results demonstrate that close spatial proximity of CD$163^{+}$ TAMs to cancer cells and the average number of CD$163^{+}$ cells either directly *adjacent* to or within *communicating* distance of each cancer cell are independent predictors of unfavorable prognosis in breast cancer.

**Abstract:**

Tumor-associated macrophages (TAMs) promote progression of breast cancer and other solid malignancies via immunosuppressive, pro-angiogenic and pro-metastatic effects. Tumor-promoting TAMs tend to express M2-like macrophage markers, including CD163. Histopathological assessments suggest that the density of CD163-positive TAMs within the tumor microenvironment is associated with reduced efficacy of chemotherapy and unfavorable prognosis. However, previous analyses have required research-oriented pathologists to visually enumerate CD$163^{+}$ TAMs, which is both laborious and subjective and hampers clinical implementation. Objective, operator-independent image analysis methods to quantify TAM-associated information are needed. In addition, since M2-like TAMs exert local effects on cancer cells through direct juxtacrine cell-to-cell interactions, paracrine signaling, and metabolic factors, we hypothesized that spatial metrics of adjacency of M2-like TAMs to breast cancer cells will have further information value. Immunofluorescence histo-cytometry of CD$163^{+}$ TAMs was performed retrospectively on tumor microarrays of 443 cases of invasive breast cancer from patients who subsequently received adjuvant chemotherapy. An objective and automated algorithm was developed to phenotype CD$163^{+}$ TAMs and calculate their density within the tumor stroma and derive several spatial metrics of interaction with cancer cells. Shorter progression-free survival was associated with a high density of CD$163^{+}$ TAMs, shorter median cancer-to-CD$163^{+}$ nearest neighbor distance, and a high number of either directly *adjacent* CD$163^{+}$ TAMs (within juxtacrine proximity <12 μm to cancer cells) or *communicating* CD$163^{+}$ TAMs (within paracrine communication distance <250 μm to cancer cells) after multivariable adjustment for clinical and pathological risk factors and correction for optimistic bias due to dichotomization.

## 1. Introduction

Despite improvements in screening, diagnosis, therapies, and surgery, breast cancer remains the second leading cause of cancer death in women [1]. The greatest reduction in breast cancer mortality would result from improved clinical management of patients diagnosed with high-risk tumors, such as those receiving chemotherapy as part of first-line treatment. While chemotherapy exerts direct cytotoxic effects on proliferating cancer cells, significant effects of chemotherapy are also mediated through immune-boosting anti-cancer mechanisms [2]. Conversely, emerging evidence from other solid tumor types indicates that strong intratumoral immunosuppressive mechanisms provide resistance to chemotherapy [3,4].

A plethora of research has demonstrated association of histological measures of anti-tumor immune activity with clinical outcome in multiple cancer types, with the main focus on tumor-infiltrating lymphocytes, particularly CD8+ cytotoxic T cells [5,6,7,8,9,10,11,12,13]. A comprehensive meta-analysis showed that the density of tumor-infiltrating lymphocytes is associated with favorable survival and predictive of pathologic complete response (pCR) in patients with HER2+ or triple-negative breast cancer (TNBC) subtypes after neoadjuvant chemotherapy [14]. In addition to the density of tumor-infiltrating lymphocytes [15], their differential spatial distribution as well as a spatial heterogeneity have prognostic value in gastrointestinal cancers [16,17,18], lung cancer [19,20,21], and breast cancer [22,23,24,25,26,27,28]. While cytotoxic T lymphocytes are tumor suppressive, tumor-associated macrophages (TAMs) exert immunosuppressive effects [29]. TAMs promote progression of breast cancer and other solid malignancies via local immunosuppressive, pro-angiogenic and pro-metastatic effects [30] that include enhancing cancer cell proliferation, invasion, and metastasis [31,32,33]. A high density of tumor-associated macrophages (TAMs) was significantly associated with poor survival in patients with breast cancer [14].

TAMs tend to display the M2-like macrophage phenotype and express CD163 [34]. While the pan-macrophage marker CD68 has been mostly used to identify TAMs in breast cancer tissues in earlier studies [35], more recent studies have established that a high density of CD$163^{+}$ TAMs in breast tumors is associated with poor prognosis [36,37,38,39]. These studies each used visual counting of CD$163^{+}$ TAMs by pathologists but employed different methodologies to support an association of poor prognosis with elevated CD$163^{+}$ TAMs in breast cancer. Medrek and colleagues relied on visual scoring of CD$163^{+}$ cells in breast cancer into 4 ordinal categories ranging from 0 (none/absent) up to 3 (strong/dense) and found that infiltration of CD$163^{+}$ macrophages into tumor stroma, but not into tumor nests, had significant association with overall survival in a cohort of 144 patients [36]. Another study reported that high levels of CD$163^{+}$ TAMs within the subjectively selected most TAM-enriched breast cancer regions were significantly associated with aggressive features, such as vessel invasion and non-luminal molecular subgroups and reduced survival in a cohort of 282 patients [39]. Jeong and colleagues found that a high number of CD$163^{+}$ macrophages within tumor nests was an independent prognostic marker of reduced OS and DFS in 367 breast cancer patients [37]. Jamiyan and colleagues reported that infiltration of CD$163^{+}$ TAMs, rather than CD68+, in both tumor stroma (TS) and tumor nests (TN) was associated with poor prognosis in patients with triple-negative breast cancer (TNBC) [38]. That study, similar to earlier reports [40,41], used methodology that involved initial identification of CD$163^{+}$ TAM “hotspot” areas (five areas of TN and five areas of TS) with most abundant macrophages followed by counting the number of CD163-positive macrophages.

These initial investigations of CD$163^{+}$ TAMs in breast cancer have largely depended on research-focused pathologists to visually enumerate the cells, which is both laborious and subjective, and impedes implementation of these analyses into clinical practice. There is a great need for objective, machine-based approaches to effectively capture the information value that can be provided by the density and spatial proximity of CD$163^{+}$ TAMs in breast cancer. Furthermore, none of the previously reported studies was focused on addressing the relationship between immunosuppressive CD$163^{+}$ TAMs in patients with high-risk breast cancer who received chemotherapy.

**Figure 1:**
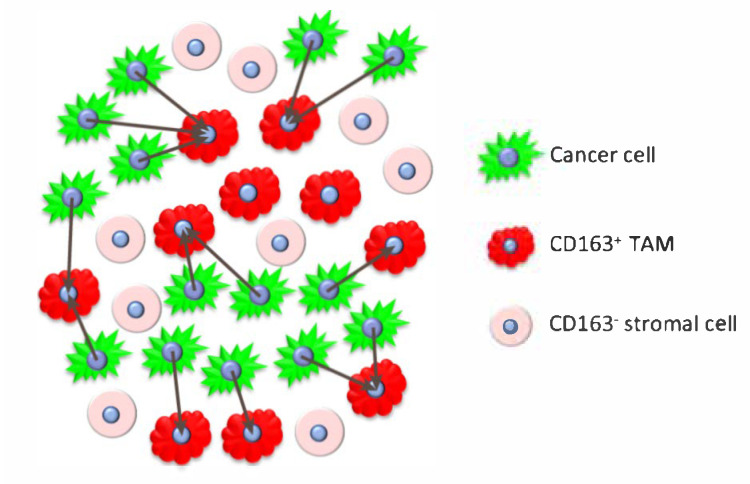
Computation of the nearest neighbor distances (NND) from each cancer cell to the nearest CD$163^{+}$ cell.

We performed immunofluorescence-based histo-cytometry of CD$163^{+}$ TAMs on tissue microarrays representing a retrospective cohort of 443 cases of invasive breast cancer from patients who subsequently received adjuvant chemotherapy. We developed objective, operator-independent image analysis methods to quantify the density of CD$163^{+}$ TAMs and explored different spatial metrics of proximity to cancer cells as predictors of clinical outcome. To objectively determine the relationship between clinical outcome and proximity of CD$163^{+}$ M2-like TAMs to breast cancer cells, three-color immunofluorescence (IF) images of breast cancer tissue microarrays were immunolabeled for cytokeratin (CK) and CD163 and counterstained with DAPI. Cell segmentation of digital images were performed by TissueStudio software (Definiens) followed by R-based procedures to provide spatial metrics as detailed in Methods and Figure 1.

## 2. Materials and Methods

### 2.1. Study Design

This retrospective study analyzed archival specimens of primary invasive breast cancer from Thomas Jefferson University Hospital, Philadelphia, PA and the Clinical Breast Care Project (CBCP) tumor archive at the Walter Reed National Military Medical Center, Bethesda, MD. All specimen and data collections were conducted according to research protocols approved by local Institutional Review Boards. Archival formalin-fixed and paraffin-embedded specimens of primary invasive breast cancer tissue specimens from 1988–2010 were collected, reviewed by central pathologist (JAH), and representative cancer tissue regions were selected for inclusion in tissue microarrays (TMAs). TMAs were constructed using the Grandmaster tissue arrayer (3DHistech) with 0.6 mm core size. The specimens from unselected consecutive breast cancer cases were included into TMAs. The patients inclusion criteria for TMA were available tumor tissue and available clinical outcome data. Missing select clinico-pathological variable(s) was not an exclusion criterion, nor were there other inclusion or exclusion criteria. The study population was further limited to patients that (i) did not have distant metastases at the time of surgery; (ii) were treated with adjuvant chemotherapy; (iii) had CD163 stained IHC images that met the quality control. A small number of patients (9) had duplicated tissue cores. For these patients, the core with the higher number of CD$163^{+}$ TAMs was used to compute CD$163^{+}$ TAM-related metrics.

### 2.2. Patient Cohort

Patients received postoperative radiotherapy according to the treating physician’s discretion. The progression-free survival (PFS) was defined as the time from diagnosis to the evidence of local, regional or distant recurrence. Only patients without distant metastatic disease at the time of diagnosis were included in the study cohort. The patients without progression were censored at the last follow-up time. Hormone receptor positivity was defined according to the definition in use at the time of diagnosis, which by immunohistochemistry ranged from ≥5% to ≥10% positivity during the study period of 1988–2010. With respect to hormone treatment, patients with HR-positive (HR+) tumors were classified as “non-compliant” if hormone treatment was recommended but patients did not receive this treatment.

### 2.3. Immunohistochemistry

Immunofluorescene immunohistochemistry (IF-IHC), slide scanning, and quantitative analysis of digitized images were performed in a manner blinded to outcome. Fluorescence immunohistochemistry for CD163 was performed on an autostainer (Agilent/Dako Omnis) using anti-CD163 antibody (Cat# HPA046404, 1:4000; Sigma) followed by HRP-conjugated secondary antibody (Cat# K4003, Agilent) and visualized using Cy5-tyramide as substrate (Cat# NEL745001KT, Perkin Elmer), multiplexed with anti-pan-cytokeratin antibody (mouse monoclonal AE1/AE3 blend, Cat# M3515, 1:100, Agilent) with Alexa-Fluor-555-labeled secondary antibody (Cat# A21422, Thermo Fisher) to identify cancer cells, followed by counterstaining with DAPI to visualize cell nuclei as previously described [42,43,44]. Stained slides were scanned at 20× magnification on the Pannoramic Flash 250 scanner (3DHistech) and fluorescent images were captured in three channels (Cy5, Cy3/Alexa-555 and DAPI). Cell segmentation of digital images was performed by TissueStudio software (Definiens) and CD163 immunoreactivity was computed for individual cancer cells and stromal cells for each tumor core. The operators were blinded to patient IDs and clinical outcomes. Some tissue cores were excluded from further analysis if the quality of the staining was deemed substandard or if less than 10 cancer cells were identified.

### 2.4. Identification of CD$163^{+}$ TAMs

The image data included spatial localization information (x-y coordinates of cell centroids) for cancer and cells within the tumor stroma. The CD163-signal in cancer cells in each tissue core were viewed as background noise and were used to compute the 99% confidence 95% content non-parametric upper tolerance limit (UTL) for background CD163 signals. The quantile cutoffs for UTLs were selected using Table A.16 in [45] with the smallest sample size of 10 and using linear interpolation for the sample sizes not included in this table. Cell within tumor stroma were identified as CD$163^{+}$ TAMs if their CD163 expression levels were above the background UTL for the corresponding tissue core. The identification procedure for CD$163^{+}$ TAMs identification was implemented in Python [46].

### 2.5. Spatial Metrics of Interaction between CD$163^{+}$ TAMs and Cancer Cells

The spatial localization of cells was jointly considered for cancer cells, CD$163^{+}$ TAMs, and CD163- stromal cells, and viewed as a multitype marked point pattern (MMPP) [47]. The resulting MMPPs were used to compute the spatial interaction metrics based on the distributions of the nearest neighbor distances (NND) between individual cancer cells and the nearest CD$163^{+}$ TAM, and CD$163^{+}$ TAM density metrics. Similar to visual enumeration of CD$163^{+}$ TAMs in a region of interest by a pathologist, we considered the total count of CD$163^{+}$ TAMs within the sampled area of each tumor tissue core (0.28 mm${}^{2}$). The proportion of CD$163^{+}$ TAMs within the stromal compartment of each tumor tissue core was also computed. Neither of these traditional metrics needs information on the spatial localization of cancer cells and immune cells. The spatial NND-based metrics included summary statistics of the NND distributions for each tissue core: (i) 10th percentile, (ii) 25th percentile (i.e., 1st quartile), and (iii) the median (50th percentile). Figure 1 illustrates the computation of the NNDs from cancer cells to their nearest CD$163^{+}$ TAM (grey arrows). In addition, we considered the average counts of *adjacent* CD$163^{+}$ TAMs to each cancer cell (within 12 μm distance of direct cell-to-cell or juxtacrine signaling) and *communicating* CD$163^{+}$ TAMs to each cancer cell (within 250 μm cyto/chemokine communication or paracrine signaling distance). The adjacent distance of 12 μm is approximately equal to the estimated median diameter of cancer cells. The choice of 250 μm is based on results of Francis and Palsson (1997) who estimated that 250 μm is the maximum tissue distance over which a single cell can effectively communicate via paracrine cyto/chemokine signaling) [48]. All spatial metrics were computed using the R package ‘spatstat’ [49]. The tumor cores that had zero count of CD$163^{+}$ TAMs (67 of the analyzed 443) were included in all analyses with the counts-based CD163 metrics equaled to zero and the NND-based metrics truncated at 600 μm, which is the diameter of the TMA tissue core.

### 2.6. Dichotomization of CD163 Metrics and Association with Clinicopathologic Characteristics and Progression-Free Survival

The correlation between CD163 metrics was evaluated using the Spearman correlation coefficient. The association between CD163 metrics and categorical clinicopathological factors was evaluated using the Kruskal–Wallis test. The optimal cutoff for each CD163 metrics with the corresponding 90% confidence interval was determined using the bootstrap approach. First, 1000 bootstrap samples were drawn with replacement from the study cohort. In each bootstrap sample, the survival tree model with 10-fold cross-validation (R package rpart [50]) was used to establish an objective data-driven optimal cutoff for dichotomizing the CD163 metric. The median of the resulting sample of 1000 cutoffs was used as the optimal cutoff for each CD163 metrics, and the corresponding non-parametric 90% confidence limits are 5th and 95th percentiles of the distribution of 1000 bootstrap-based cutoffs. The association between dichotomized CD163 metrics and categorical clinicopathological factors was evaluated using the Fisher’s exact test or its extension for more than 2 categories. For the continuous age variable, the two-sample Wilcoxon Signed-Rank test was used. The *p*-values for testing associations between multiple continuous or dichotomized CD163 metrics and clinicopathological factors were adjusted for multiple testing using Holm’s method which controls family-wise error rate [51].

Each CD163 metric computed was evaluated as a dichotomized predictor of PFS in a univariate Cox model and multivariable Cox model that included standard clinicopathological prognostic factors of PFS: age, race (white vs. non-white), histologic grade, node status, tumor size (<2 cm, 2–5 cm, >5 cm), radiation therapy, chemotherapy, and hormone therapy non-compliance (vs. the compliant reference category). The parsimonious model was selected a priori including clinicopathological prognostic factors but not the CD163 markers. The proportional hazards assumption was evaluated for all covariates in the Cox models and covariates that violated the proportional hazard assumptions were incorporated in the models as strata variables.

### 2.7. Internal Validation of Dichotomized CD163 Metrics

The internal validation of dichotomized CD$163^{+}$ metrics was performed using the bootstrap optimism correction procedure [52]. Multiple imputation was used for missing values for clinicopathologic covariates for some patients (0.6% to 10% for all clinicopathological covariates, but higher level for hormone compliance, Table 1, Supplementary Tables S2–S7). Forty (40) imputed datasets were created using the multivariate imputation by chained equations (MICE) algorithm. For each covariate, missing values were imputed by univariate models for corresponding outcome type using the fully conditional specification [53]. The bootstrap optimism correction algorithm was applied to each imputed data set. Then results for all imputed data sets were averaged using Rubin’s rule [54]. The following steps of the bootstrap optimism correction algorithm were performed for each imputed data set. First, 500 bootstrap samples were drawn with replacement from each imputed data set with all patients. In each bootstrap sample, the survival tree model with 10-fold cross-validation (R package rpart) was used to establish an objective data-driven optimal cutpoint for dichotomizing the CD163 metric. The cutpoint from the current bootstrap sample was used to compute the log hazard ratio for the univariate and multivariate Cox models in the current bootstrap sample (“Bootstrap performance”) and in the main sample (“Test performance”), and the optimism in hazard ratio estimation was computed as the difference between log hazard ratio for “Bootstrap performance” and for “Test performance”. The mean optimism estimate was computed as the average optimism over 500 bootstrap samples. The cutpoint for dichotomizing individual spatial CD163 metrics was also established in the imputed data set and its “apparent performance” was computed as the log hazard ratio for dichotomized CD163 metric in the multivariable Cox models. Finally, the optimism-corrected performance estimates were computed by subtracting the mean optimism estimates from the apparent performance estimates [52]. The optimal cutpoints based on bootstrap samples were used to compute bootstrap-based non-parametric 90% confidence intervals for dichotomization cutpoints. The outcome and association analyses were performed using R [55].

## 3. Results

The study cohort included 443 cases of locoregional HR+ and HR- breast cancer treated with adjuvant chemotherapy for whom tissue sample included at least 10 cancer cells. The clinical follow-up ranged from 1 month to 238 months with a median follow-up time of 64 months. There were 77 progressions in the study cohort of 443 patients (17.4%). Table 1 details the patient characteristics and their association with the dichotomized standard metric of CD$163^{+}$ TAM count per tissue core.

**Figure 2:**
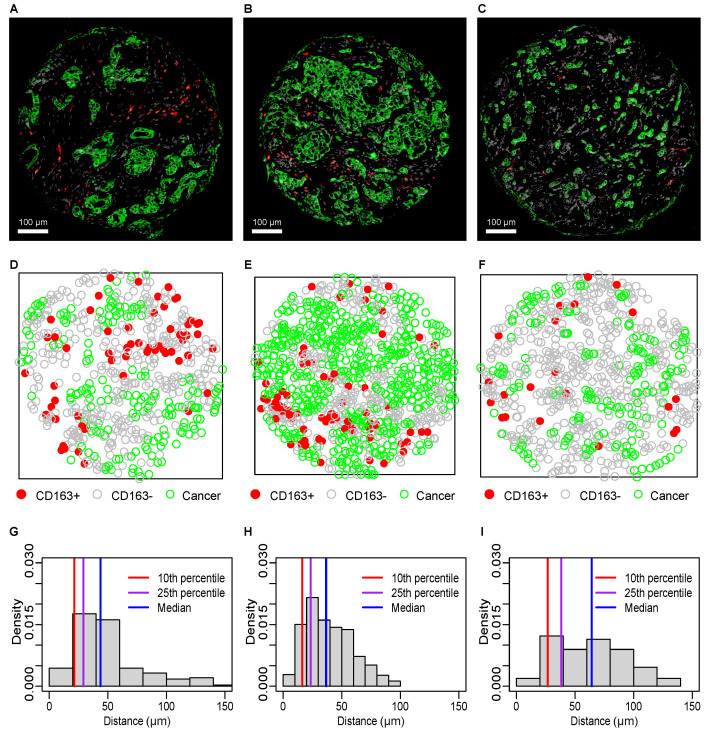
Spatial analysis of CD$163^{+}$ TAMs and cancer cells in breast cancer. IF-IHC images ((**A**–**C**); cancer cells green, CD$163^{+}$ TAMs red) with corresponding marked point patterns (**D**–**F**) of CD$163^{+}$ TAMs (red), $CD163^{-}$ cells (grey), and cancer cells (green), and nearest neighbor distance (NND) distributions (**G**–**I**) for cancer cells to the nearest CD$163^{+}$ TAM.

Examples of breast cancer tissue core IF-IHC images are shown in Figure 2A–C with the corresponding multitype marked point patterns (MMPPs) of cancer cells (green), CD$163^{+}$ TAMs (red), and CD$163^{-}$ stromal cells (gray; Figure 2D–F), and the corresponding distributions of nearest neighbor distances (NNDs) from cancer cells to the nearest CD$163^{+}$ TAM with metrics of 10th percentile, 25th percentile (lower quartile Q1) and median NNDs (Figure 2G–I).

For the tumor core presented in Figure 2B, the NND distribution from cancer cells to CD$163^{+}$ TAMs (Figure 2H) is reflective of greater proximity (smaller range and smaller values of all percentiles, including 10th, 25th and median) than the NND distributions in Figure 2G,I, which corresponds to the tumor cores in Figure 2A,C. Such more proximal distribution corresponds to a higher prevalence of CD$163^{+}$ TAMs found next to cancer cells in MMPP (Figure 2E). In contrast, the majority of CD$163^{+}$ TAMs in Figure 2D,F are more separated of from cancer cells and the corresponding NND distributions in Figure 2G,I are wider and have larger values of all percentiles, including the 10th and 25th percentile and the median. As described in the Methods section, we also computed alternative spatial proximity metrics of the average number of CD$163^{+}$ TAMs that are (i) directly *adjacent* to (within 12 μm distance or (ii) within paracrine cyto/chemokine *communicating* distance (within 250 μm distance) [48] of each cancer cell.

We first examined the correlations among all CD$163^{+}$ TAM-related spatial metrics, including count and proportion of CD$163^{+}$ TAMs, cancer-to-CD$163^{+}$ NND-based metrics, and metrics of CD$163^{+}$ TAM proximity to cancer cells (average numbers of *adjacent* or *communicating* CD$163^{+}$ TAMs) (Supplementary Figure S1 and Supplementary Table S1). As expected, the traditional count of CD$163^{+}$ TAMs in the tumor core and the proportion of CD$163^{+}$ TAMs among stromal cells were highly correlated (coefficient = 0.74; 95% CI: 0.69; 0.78). The average number of *communicating* CD$163^{+}$ TAMs was highly correlated with CD$163^{+}$ TAM count (coefficient = 0.989; 95% CI: 0.987; 0.991) and stromal cell CD$163^{+}$ TAM proportion (coefficient = 0.76; 95% CI: 0.71; 0.8) (Supplementary Table S1). The three NND metrics were highly pairwise correlated, but not as correlated with the count and proportion or with the metrics of CD$163^{+}$ TAM proximity to cancer cells (Supplementary Figure S1). Meanwhile, the two metrics of CD$163^{+}$ TAM proximity to cancer cells were substantially correlated (coefficient = 0.55; 95% CI: 0.49; 0.62) (Supplementary Table S1).

Supplementary Figures S2 and S3 present the associations between all CD$163^{+}$ TAM-related metrics and clinicopathologic characteristics. Only *p*-values significant after multiple testing adjustment are shown on the plots. Notably, HR-negative (HR-) tumors had significantly higher levels of traditional CD$163^{+}$ TAM count-based metrics and a higher average number of *communicating* CD$163^{+}$ TAMs as compared patients with HR-positive (HR+) tumors, but no difference between HR- and HR+ tumors was observed in terms of the NND-based CD$163^{+}$ TAM metrics or the average number of *adjacent* CD$163^{+}$. Similarly, higher levels of CD$163^{+}$ TAM count-based metrics were associated with higher histologic grade, but no such trend was observed for the NND-based metrics. However, NND-based CD$163^{+}$ TAM metrics were higher in Her2-positive as compared to Her2-negative tumors.

We next explored dichotomization for all CD$163^{+}$ TAM-related metrics. Optimal dichotomization thresholds were identified for each CD$163^{+}$ TAM-related metric, and all dichotomized count-based and distance-based metrics remained significant predictors of progression-free survival (PFS) after bootstrap-based bias correction and adjustment for multiple testing (Table 2). Table 1 and Supplementary Tables S2–S8 present the associations of all dichotomized CD$163^{+}$ TAM metrics with the standard clinicopathological prognostic factors of PFS and adjuvant treatment indicators. Similar to results for continuous CD$163^{+}$ TAM metrics, HR- breast tumors have higher proportions of cases with a high total count of CD$163^{+}$ TAMs and a high number of *adjacent* or *communicating* CD$163^{+}$ TAMs as compared HR+ tumors (Table 1 and Supplementary Tables S6 and S7). Also, high levels of all count-based CD$163^{+}$ TAM metrics were associated with higher histological grade (Table 1 and Supplementary Tables S2, S6 and S7), and high CD$163^{+}$ TAM NND-based metric were associated with higher proportion of Her2-positive tumors (Supplementary Tables S3–S5). No association with age was observed for any of the CD$163^{+}$ TAM metrics (Supplementary Table S8).

The highest bias-adjusted effect sizes (hazard ratios) on PFS were observed for the count of CD$163^{+}$ TAMs per core (HR = 3.0; 95% CI: 1.7–5.1; *p* < 0.001) and the number of *communicating* CD$163^{+}$ TAMs (HR = 3.0; 95% CI: 1.8—4.9; *p* < 0.001), followed by the number of CD$163^{+}$ TAMs *adjacent* to cancer cells (HR = 2.7; 95% CI: 1.7–4.3; *p* < 0.001). These CD$163^{+}$ TAM metrics as well as the median of the cancer cell-to-CD$163^{+}$ TAM NND distribution remained significant predictors of PFS in multivariable Cox models adjusted for significant clinicopathological prognostic factors (Table 3). Each CD$163^{+}$ TAM metric was evaluated in separate multivariable Cox model due to high correlations between these metrics (Supplementary Table S1). The bias-adjusted effect sizes for all CD$163^{+}$ TAM metrics in multivariable Cox models are presented in Supplementary Table S9.

**Figure 3:**
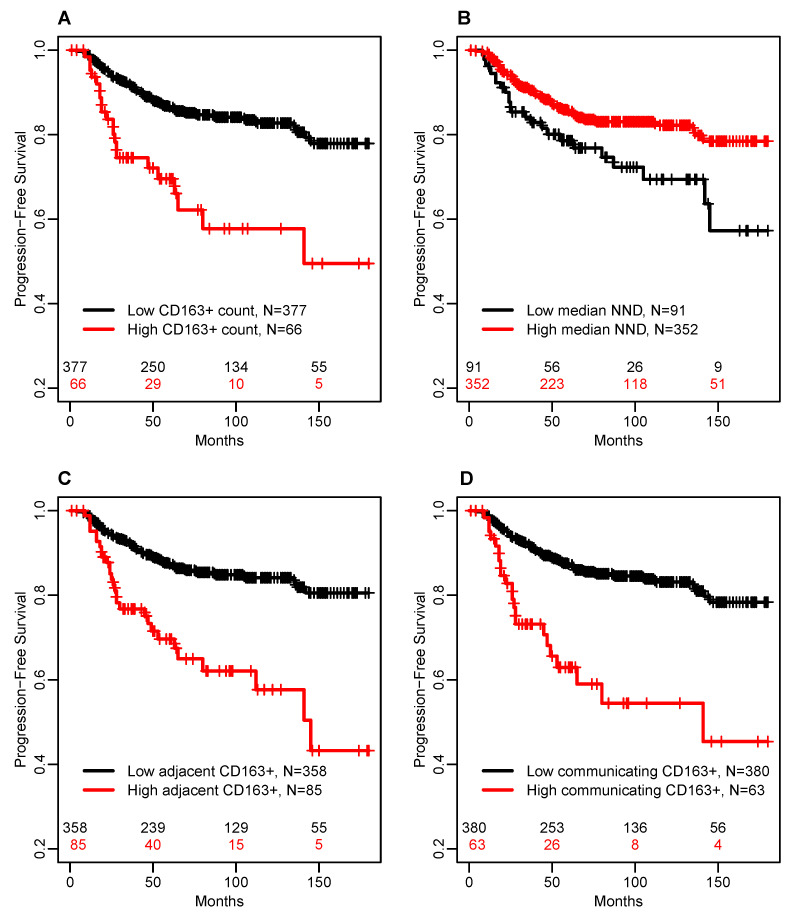
Kaplan–Meier plots of PFS by CD163-related markers with the highest prognostic value in the multivariable Cox model adjusted for significant clinicopathologic risk factors. (**A**) total number of CD$163^{+}$ TAMs in the sampled tumor area (CD$163^{+}$ Count); (**B**) median nearest neighbor distance (median NND) from cancer cells to CD$163^{+}$ TAMs; (**C**) the average number of CD$163^{+}$ TAMs within 12 μm distance of each cancer cell (adjacent CD$163^{+}$); (**D**) the average number of CD$163^{+}$ TAMs within 250 μm distance of each cancer cell (communicating CD$163^{+}$).

In multivariable Cox models adjusted for significant clinicopathological prognostic factors of PFS, the number of *communicating* CD$163^{+}$ TAMs had the largest effect size (HR = 2.3; 95% CI: 1.4–4.0; *p* = 0.003), followed by the median of the CD$163^{+}$ TAM NND distribution (HR = 1.9; 95% CI: 1.03–3.4; *p* = 0.024), the count of CD$163^{+}$ TAMs per core (HR = 1.9; 95% CI: 1.05–3.4; *p* = 0.039), and the number of *adjacent* CD$163^{+}$ TAMs (HR = 1.8; 95% CI: 1.1–2.9; *p* = 0.024). For illustration, Figure 3 shows the Kaplan–Meier survival estimates for these CD$163^{+}$ TAM metrics dichotomized using the thresholds obtained as the median threshold in 1000 bootstrap samples (Table 2).

**Figure 4:**
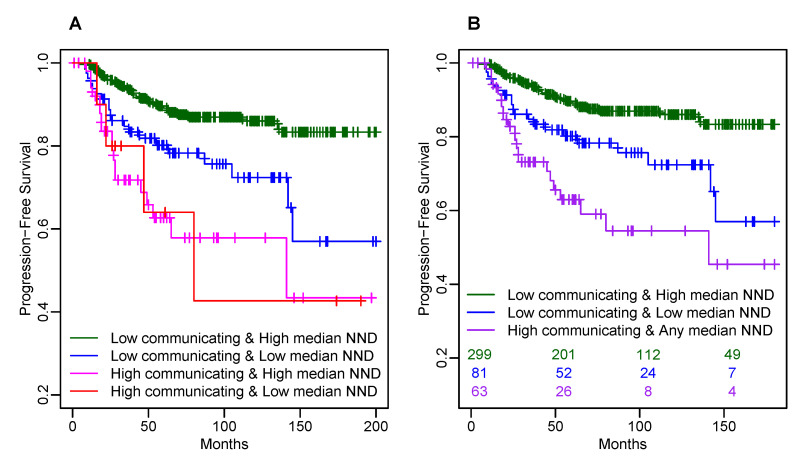
Kaplan–Meier plots of PFS by combined CD$163^{+}$ TAM marker based on high vs. low average number of *communicating* CD$163^{+}$ cells and high vs. low median NND. (**A**) by four combinations of low/high average number of *communicating* CD$163^{+}$ cells and low/high median cancer-to-CD$163^{+}$ NND; (**B**) by combined CD163 marker with 3 categories: (1) low average number of *communicating* CD$163^{+}$ cells and high median cancer-to-CD$163^{+}$ NND; (2) low average number of *communicating* CD$163^{+}$ cells and low median cancer-to-CD$163^{+}$ NND; (3) high average number of *communicating* CD$163^{+}$ cells and any median cancer-to-CD$163^{+}$ NND.

The count-based CD$163^{+}$ TAM metrics significant in multivariable Cox models were highly correlated (correlation coefficients ranging from 0.53 to 0.99, Supplementary Table S1), but the median cancer-to-CD$163^{+}$ NND was less correlated with all these count-based CD$163^{+}$ TAM metrics (correlation coefficients ranging from −0.12 to −0.33; Supplementary Table S1). Thus, for the secondary analysis, we considered combining the strongest count-based predictor (the number of *communicating* CD$163^{+}$ TAMs) with the median cancer-to-CD$163^{+}$ NND. The model with the main effects and interaction between these two metrics did not yield a significance of interaction term most likely due to the sample size limitation. However, Kaplan–Meier survival estimates of four combinations of low/high number of *communicating* CD$163^{+}$ TAMs and low/high median cancer-to-CD$163^{+}$ NND (Figure 4A) suggested possibly more than 2 different risk categories based on the dichotomized number of *communicating* CD$163^{+}$ TAMs and the median cancer-to-CD$163^{+}$ NND. Therefore, a combined CD$163^{+}$ TAM marker was defined with three risk categories: (1) low number of *communicating* CD$163^{+}$ TAMs and high median cancer-to-CD$163^{+}$ NND; (2) low number of *communicating* CD$163^{+}$ TAMs and low median cancer-to-CD$163^{+}$ NND; (3) high number of *communicating* CD$163^{+}$ TAMs and any median cancer-to-CD$163^{+}$ NND. Figure 4B shows for illustration the corresponding unadjusted Kaplan–Meier survival estimates for the resulting three groups. Table 4 shows the results from the multivariable Cox model including the combined CD$163^{+}$ TAM marker and significant clinicopathological prognostic factors with bootstrap-based optimism correction. This model suggests that with adjustment for known significant clinicopathological prognostic factors, similar extra risk of progression is associated with a high number of *communicating* CD$163^{+}$ TAMs (HR = 2.5; 95% CI: 1.4–4.9; *p* = 0.002) and with low median cancer-to-CD$163^{+}$ NND (HR = 2.3; 95% CI: 1.1–4.6; *p* = 0.024) as compared to the lowest risk group with a low number of *communicating* CD$163^{+}$ cells and high median cancer-to-CD$163^{+}$ NND (Table 4). The unadjusted Kaplan–Meier survival estimates for the resulting three groups suggest higher risk associated with a high number of *communicating* CD$163^{+}$ TAMs than with how median cancer-to-CD$163^{+}$ NND because of association between hormone receptor status and combined CD$163^{+}$ TAM marker. Namely, 33/63 (52%) of tumors with a high number of *communicating* CD$163^{+}$ TAMs are HR-, but only 12/81 (15%) of tumors with a low number of *communicating* CD$163^{+}$ TAMs and low median cancer-to-CD$163^{+}$ NND are HR-. Thus, adjustment for hormone receptor status reduces the effect size for a high number of *communicating* CD$163^{+}$ TAMs in the multivariable Cox model. Meanwhile, the effect of low median cancer-to-CD$163^{+}$ NND is attributed primarily to HR+ tumors overrepresented in the risk group with low median cancer-to-CD$163^{+}$ NND and a low number of *communicating* CD$163^{+}$ TAMs.

## 4. Discussion

Mounting evidence indicates that high levels of tumor-associated macrophages (TAMs) [6,25,36,56], and especially CD$163^{+}$ TAMs of the M2-like phenotype [34,37,38,39], are associated with poor prognosis in breast cancer. However, the published evidence has largely centered on pathologists’ visual enumeration of CD$163^{+}$ TAMs with limited capability to objectively capture prognostic value embedded in the spatial relationship between of CD$163^{+}$ TAMs and cancer cells. In this study, we developed a novel method for objective phenotyping of CD$163^{+}$ TAMs and present new spatial metrics of proximity of CD$163^{+}$ TAMs to cancer cells that are independent predictors of clinical outcome in high-risk breast cancer patients treated with adjuvant chemotherapy. The new algorithm identifies CD$163^{+}$ TAMs in an objective manner, independent of subjective assessment by trained pathologists or by subjective thresholding by the operator of the image analysis software. The spatial interactions between cancer cells and CD$163^{+}$ TAMs are quantified objectively using the spatial metrics based on nearest neighbor distance (NND) distribution and the average number of CD$163^{+}$ cells either directly in contact with cancer cells (adjacent within 12 μm of cancer cells) or within paracrine signaling distance of cancer cells (communicating within <250 μm distance from cancer cells).

Since the circles with 250 μm radius are comparable in size with the tissue core with diameter of 600 μm, the CD$163^{+}$ TAM count and the average count of *communicating* CD$163^{+}$ TAMs are highly correlated in our study. However, the average number of *communicating* CD$163^{+}$ TAMs has the advantage that this metric can be applied directly to histological images of any size (whole tissue, biopsy or TMA) without the need to select a tumor region of interest.

Moreover, our results indicate that the average number of *communicating* CD$163^{+}$ TAMs is a potentially more informative metric than the traditional density of CD$163^{+}$ TAMs within a sampled area of the tumor. Similarly, all NND-based metrics and the average number of *adjacent* CD$163^{+}$ TAMs can be used in tumor images of any size and can also be computed for other immune cell populations and can be applied to other solid tumor types. Although we focused on the analysis of breast cancer data, higher CD$163^{+}$ TAM density has been shown to be correlated with worse prognosis in other cancer subtypes such as cutaneous melanoma [57], colorectal carcinoma [58,59], and ovarian carcinoma [60]. These studies primarily focused on the density of TAMs rather than spatial arrangements of TAMs. However, some studies showed that increased CD$163^{+}$ TAM prevalence was associated with improved clinical outcomes in colorectal carcinoma [61,62]. Thus, as pointed out by Krijgsman et al. [63], it is critical to characterize the spatial distribution of TAMs in order to fully understand the association between TAMs and clinical outcomes.

Our studies of the spatial distribution of CD$163^{+}$ TAMs differ from previously described quantitative analyses of TAMs in tumor microenvironment in several aspects. First, virtually all reported studies used trained pathologists to analyze tumor specimens, while we developed methodology which allowed for the operator-independent identification of cancer cells and CD$163^{+}$ TAMs in the tumor stroma. Second, we developed a novel method for objective phenotyping of CD$163^{+}$ TAMs that does not rely on subjective identification of CD$163^{+}$ TAMs. Third, we present spatial metrics that have not been used in other studies of CD$163^{+}$ TAMs in the tumor microenvironment, although NND-based metrics have been investigated for CD8+ T cells in solid tumors in multiple reports.

The role of tumor-infiltrating lymphocytes (TILs) has been an active area of breast cancer research [5,6,7,8,9,10,11,12,13]. There is a potential for association between metrics based on TILs and TAMs, but such investigation is beyond the scope of this paper, and will be a subject of further research. Another limitation of our study is that we do not consider the association between PD-L1 status and CD$163^{+}$ TAM metrics. Limitations of this study also include the retrospective analysis of archived tissue samples and the use of TMAs, which provide limited sampling of each tumor. For a majority of the patients, only one core was included into the TMA. Our results were internally validated using rigorous statistical analysis, but validation in independent patient cohort(s) and whole-tissue sections will be needed. However, more extensive tumor sampling from whole-tissue sections is expected to provide more accurate data and potentially strengthen the results based on spatial CD$163^{+}$ TAM metrics from limited TMA cores.

## 5. Conclusions

Our results demonstrate for the first time that close spatial proximity of CD$163^{+}$ TAMs to cancer cells and the average number of *adjacent* or *communicating* CD$163^{+}$ TAMs are independent predictors of unfavorable prognosis in breast cancer. In breast cancer patients who received chemotherapy, we found that the average number of *communicating* CD$163^{+}$ TAMs had the largest effect size (hazard ratio) in the multivariable Cox model for PFS adjusted for known significant clinicopathological risk factors and corrected for optimistic bias associated with dichotomization. However, the confidence intervals of this hazard ratio largely overlap with similarly adjusted hazard ratios for the dichotomized median cancer-to-CD$163^{+}$ NND or the average number of *adjacent* CD$163^{+}$ TAMs. Hence, it remains possible that the spatial metrics of median cancer-to-CD$163^{+}$ NND or the number of *adjacent* CD$163^{+}$ TAMs may become more important predictors than the number of *communicating* CD$163^{+}$ cells in other solid tumors or in distinct breast cancer cohorts, for instance in different breast cancer subtypes or under different treatment.

Methods to identify breast cancer patients with an elevated risk profile based on CD$163^{+}$ TAM-centered tumor markers have the potential to improve clinical management of patients by identifying patients with unfavorable prognosis and opening the way for alternative tumor immune-boosting strategies. The present study builds on past progress of cell density-based tumor analyses and provides new insights into the prognostic significance of spatial proximity of CD$163^{+}$ TAMs to cancer cells using objective and quantitative methodologies. The described automated analysis pipeline is readily applicable to both core needle biopsies and whole-tissue specimens and is expected to be relevant beyond breast cancer. The prognostic values of CD$163^{+}$ TAM density and spatial proximity to breast cancer cells may also be independent prognostic factors in other solid malignancies.

## Figures and Tables

**Table 1:** Associations between dichotomized count of CD163+ cells and other prognostic factors.

		Number of CD163+ Cells per 0.28 mm${}^{2}$				
			Low		High	
	Total N	N	%	N	%	*p (&)*
	443	377	85%	66	15%	
HR-	93	62	67%	31	33%	<0.001
HR+	349	314	90%	35	10%	
Missing	1	1	100%	0	0%	
White	101	81		20	20%	1.000
Non-White	333	288	86%	45	14%	
Unknown	9	8	89%	1	11%	
Histological Grade 1	119	109	92%	10	8%	0.018
Histological Grade 2	177	157	89%	20	11%	
Histological Grade 3	145	109	75%	36	25%	
Missing	2	2	100%	0	0%	
Tumor Size <2 cm	214	191	89%	23	11%	1.000
Tumor Size 2–5 cm	171	136	80%	35	20%	
Tumor Size >5 cm	58	50	86%	8	14%	
Node Negative	211	179	85%	32	15%	1.000
Node Positive	223	190	85%	33	15%	
Missing	9	8	89%	1	11%	
Her2 Negative	375	321	86%	54	14%	1.000
Her2 Positive	54	44	81%	10	19%	
Missing	14	12	86%	2	14%	
Radiation = Yes	255	219	86%	36	14%	1.000
Radiation = No	188	158	84%	30	16%	
Horm Tx Non-Compliant	98	89	91%	9	9%	0.003
Horm Tx Compliant	45	40	89%	5	11%	
Unknown	207	186	90%	21	10%	

(*&*) adjusted for multiple testing using Holm’s method.

**Table 2:** Bias-adjusted hazard ratios and optimal cutpoints for dichotomized metrics.

Metric	Dichotomization Threshold (#)	Hazard Ratio (*)	LL 95%CI	UL 95%CI	*p (&)*
High vs. low number of CD$163^{+}$ TAMs	70 (12–102)	2.97	1.73	5.11	<0.001
High vs. low proportion of CD$163^{+}$ TAMs	0.038 (0.004–0.149)	1.94	1.20	3.14	0.026
High vs. low 10th PCTL of cancer-to-CD$163^{+}$ NNDs	13 (7–55)	0.60	0.38	0.94	0.027
High vs. low 25th PCTL of cancer-to-CD$163^{+}$ NNDs	17 (9–42)	0.46	0.26	0.82	0.026
High vs. low median cancer-to-CD$163^{+}$ NND	19 (13–74)	0.48	0.27	0.85	0.026
High vs. low average number of *adjacent* CD$163^{+}$ TAMs	0.034 (0.022–0.087)	2.71	1.70	4.32	<0.001
High vs. low average number of *communicating* CD$163^{+}$ TAMs	46 (21–57)	2.96	1.80	4.87	<0.001

(#) bootstrap-based 90% confidence interval in parentheses. (*&*) adjusted for multiple testing using Holm’s method. (*) bootstrap-based optimistic bias-adjusted hazard ratio.

**Table 3:** Bias-adjusted results from the parsimonious Cox models with significant CD163 metrics.

Model with Number of CD$163^{+}$ TAMs	Hazard Ratio (*)	LL 95% CI	UL 95% CI	*p*-Value
Histological Grade 2 vs. 1	2.01	0.92	4.39	0.086
Histological Grade 3 vs. 1	2.42	1.05	5.58	0.042
HR- vs. HR+ Hormone Tx compliant	1.52	0.78	3.00	0.226
HR+ Hormone Tx Non-Compliant vs. Compliant	2.74	1.43	5.24	0.004
Node Positive vs. Negative	1.84	1.10	3.07	0.022
Tumor Size 2–5 cm vs. <2 cm	1.96	1.15	3.36	0.016
Tumor Size >5 cm vs. <2 cm	2.93	1.53	5.61	0.002
High vs. Low Number of CD$163^{+}$ TAMs	1.89	1.05	3.41	0.039
**Model with Median Cancer-to-CD$163^{+}$ NND**	Hazard Ratio (*)	**LL 95% CI**	**UL 95% CI**	***p*** **-Value**
Histological Grade 2 vs. 1	2.35	1.08	5.11	0.036
Histological Grade 3 vs. 1	2.87	1.26	6.53	0.015
HR- vs. HR+ Hormone Tx Compliant	1.90	1.00	3.61	0.055
HR+ Hormone Tx Non-Compliant vs. Compliant	2.71	1.39	5.28	0.005
Node Positive vs. Negative	1.78	1.06	2.97	0.032
Tumor Size 2–5 cm vs. <2 cm	1.85	1.08	3.17	0.029
Tumor Size >5 cm vs. <2 cm	2.76	1.44	5.30	0.003
Low vs. High Median Cancer-to-CD$163^{+}$ NND	1.88	1.03	3.44	0.024
**Model with Average Number of** ***Adjacent*** **$\mathrm{CD}163^{+}$ TAMs**	Hazard Ratio (*)	**LL 95% CI**	**UL 95% CI**	***p*** **-Value**
Histological Grade 2 vs. 1	1.99	0.91	4.36	0.090
Histological Grade 3 vs. 1	2.18	0.94	5.04	0.074
HR- vs. HR+ Hormone Tx Compliant	1.71	0.89	3.29	0.111
HR+ Hormone Tx Non-Compliant vs. Compliant	2.61	1.37	4.97	0.005
Node Positive vs. Negative	1.84	1.11	3.07	0.022
Tumor Size 2–5 cm vs. <2 cm	1.97	1.15	3.36	0.016
Tumor Size >5 cm vs. <2 cm	2.76	1.44	5.28	0.003
High vs. Low Average Number of *adjacent* CD$163^{+}$ TAMs	1.78	1.09	2.89	0.024
**Model with Average Number of** ***Communicating*** **$\mathrm{CD}163^{+}$ TAMs**	Hazard Ratio (*)	**LL 95% CI**	**UL 95% CI**	***p*** **-Value**
Histological Grade 2 vs. 1	2.25	1.03	4.92	0.045
Histological Grade 3 vs. 1	2.85	1.24	6.57	0.016
HR- vs. HR+ Hormone Tx Compliant	1.48	0.75	2.91	0.267
HR+ Hormone Tx Non-Compliant vs. Compliant	2.55	1.32	4.92	0.008
Node Positive vs. Negative	1.79	1.08	2.97	0.027
Tumor Size 2–5 cm vs. <2 cm	1.81	1.06	3.09	0.034
Tumor Size >5 cm vs. <2 cm	2.77	1.45	5.28	0.003
High vs. Low Average Number of *communicating* CD$163^{+}$ TAMs	2.33	1.37	3.98	0.003

(*) Bootstrap-based optimistic bias-adjusted hazard ratio.

**Table 4:** Bias-adjusted results from the parsimonious Cox models with combined CD$163^{+}$ TAM marker.

Predictor	Hazard Ratio (*)	LL 95% CI	UL 95% CI	*p*-Value
Histological Grade 2 vs. 1	2.17	0.99	4.76	0.056
Histological Grade 3 vs. 1	2.74	1.19	6.31	0.021
HR- vs. HR+ Hormone Tx Compliant	1.52	0.77	3.01	0.233
HR+ Hormone Tx Non-Compliant vs. Compliant	2.67	1.35	5.28	0.007
Node Positive vs. Negative	1.86	1.12	3.09	0.019
Tumor Size 2–5 cm vs. <2 cm	1.75	1.02	3.00	0.046
Tumor Size >5 cm vs. <2 cm	2.81	1.48	5.33	0.002
Low vs. High Median Cancer-to-CD$163^{+}$ NND AND Low Average Number of *communicating* CD$163^{+}$ TAMs	2.29	1.14	4.63	0.024
High vs. Low Average Number of *communicating* CD$163^{+}$ TAMs AND High or Low Median Cancer-to-CD$163^{+}$ NND	2.47	1.42	4.29	0.002

(*) Bootstrap-based optimistic bias-adjusted hazard ratio.

## Data Availability

The data sets used and/or analyzed during the current study are available from the corresponding authors on request.

## Supplementary Materials

The following are available online at https://www.mdpi.com/article/10.3390/cancers14020308/s1, Figure S1: Scatter plots between all pairs of CD163-related markers. Figure S2: Boxplots of count-based CD$163^{+}$ TAM-related metrics by standard clinicopathologic characteristics., Figure S3: Boxplots of NND-based CD$163^{+}$ TAM-related metrics by standard clinicopathologic characteristics. Table S1: Correlation coefficients with the corresponding 95% confidence intervals. Table S2: Associations between dichotomized stromal proportion of CD$163^{+}$ cells and other prognostic factors. Table S3: Associations between dichotomized 10th percentile of the NND distribution and other prognostic factors. Table S4: Associations between dichotomized 25th percentile of the NND distribution and other prognostic factors. Table S5: Associations between dichotomized median of the NND distribution and other prognostic factors. Table S6: Associations between the dichotomized number of CD$163^{+}$ cells within 12 μm from cancer and other prognostic factors. Table S7: Associations between the dichotomized number of CD$163^{+}$ cells within 250 μm from cancer and other prognostic factors. Table S8: Associations between age at diagnosis and dichotomized CD163 markers. Table S9: Bias-adjusted hazard ratios from the multivariate parsimonious Cox models with imputations and including dichotomized CD163 metrics one at a time.

## Abbreviations

The following abbreviations are used in this manuscript:

CI	Cyonfirdence interval
CK	Cytokeratin
IHC	Immunohistorychemistry
LL	Lower limit
MICE	Multivariate imputation by chained equations
MMPP	Multitype marked point patterns
NND	Nearest neighbor distance
pCR	Pathologic complete response
PCTL	Percentile
PFS	Progression-free survival
TAM	Tumor-associated macrophages
TIME	Tumor immune microenvironment
TMA	Tissue microarray
TN	Tumor nest
TNBC	Triple-negative breast cancer
TS	Tumor stroma
UL	Upper limit
UTL	Upper tolerance limit
