# Supplementary material for: Spatial Metrics of Interaction between CD163-Positive Macrophages and Cancer Cells and Progression-Free Survival in Chemo-Treated Breast Cancer
Source: Cancers (Basel). 2022 Jan 8;14(2):308. doi: 10.3390/cancers14020308 (PMC8773496; doi:10.3390/cancers14020308)
Supplement: Supplementary file 1 [file cancers-14-00308-s001.zip › SupplFigure S3.pdf]

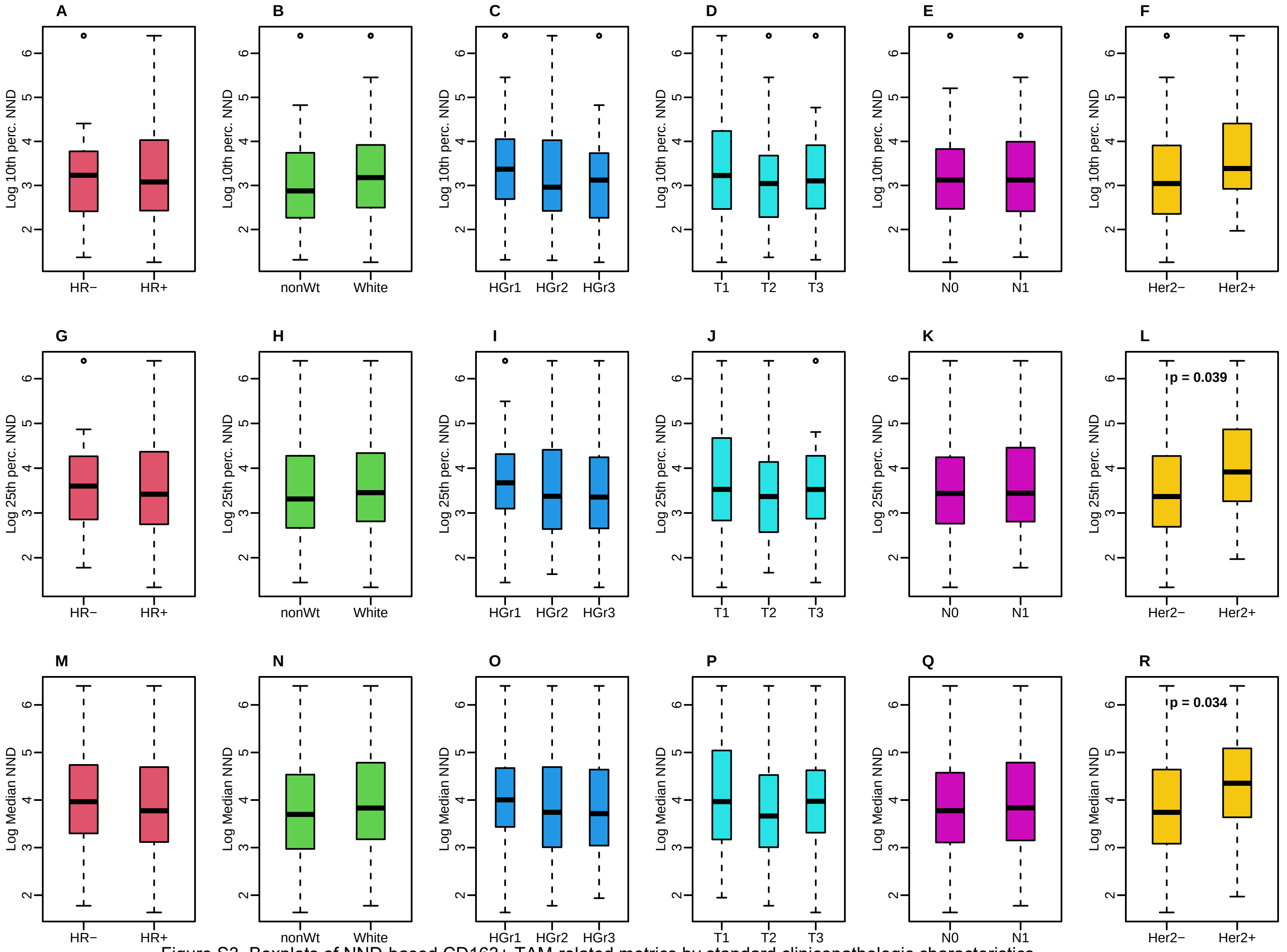

Figure S3. Boxplots of NND-based CD163+ TAM-related metrics by standard clinicopathologic characteristics.
